# Liquid Biopsy Landscape in Patients with Primary Upper Tract Urothelial Carcinoma

**DOI:** 10.3390/cancers14123007

**Published:** 2022-06-18

**Authors:** Stephanie N. Shishido, Alireza Ghoreifi, Salmaan Sayeed, George Courcoubetis, Amy Huang, Brandon Ye, Sankalp Mrutyunjaya, Inderbir S. Gill, Peter Kuhn, Jeremy Mason, Hooman Djaladat

**Affiliations:** 1 Convergent Science Institute in Cancer, Michelson Center for Convergent Bioscience, University of Southern California, Los Angeles, CA 90089, USA; sshishid@usc.edu (S.N.S.); salmaans@usc.edu (S.S.); courcoub@usc.edu (G.C.); awhuang@usc.edu (A.H.); branye@usc.edu (B.Y.); mrutyunj@usc.edu (S.M.); 2 Catherine and Joseph Aresty Department of Urology, Institute of Urology, Keck School of Medicine, University of Southern California, Los Angeles, CA 90033, USA; alireza.ghoreifi@med.usc.edu (A.G.); igill@med.usc.edu (I.S.G.); djaladat@med.usc.edu (H.D.); 3 Norris Comprehensive Cancer Center, Keck School of Medicine, University of Southern California, Los Angeles, CA 90033, USA; 4 Department of Biological Sciences, Dornsife College of Letters, Arts, and Sciences, University of Southern California, Los Angeles, CA 90089, USA

**Keywords:** upper tract urothelial carcinoma, liquid biopsy, HDSCA, circulating tumor cell, large extracellular vesicle, peripheral blood

## Abstract

**Simple Summary:**

The standard of care for patients diagnosed with upper tract urothelial carcinoma (UTUC) is surgery. However, the current standard tools for the diagnosis and preoperative risk stratification of primary UTUC leave much to be desired. Using a simple blood sample, we can detect rare events, such as circulating tumor cells (CTCs) and large extracellular vesicles (LEVs), which may reveal biomarkers indicative of disease and provide insight into progression. This study demonstrates a wide variety of CTCs and LEVs that are identifiable in the blood of patients with UTUC at higher counts compared to normal donors. The data presented indicate that a blood-based liquid biopsy can detect UTUC and help guide clinical decisions.

**Abstract:**

Urothelial carcinomas (UCs) are a broad and heterogeneous group of malignancies, with the prevalence of upper tract urothelial carcinoma (UTUC) being rare, accounting for only 5–10% of total malignancies. There is a need for additional toolsets to assist the current clinical paradigm of care for patients with UTUC. As a non-invasive tool for the discovery of cancer-related biomarkers, the liquid biopsy has the potential to represent the complex process of tumorigenesis and metastasis. Herein, we show the efficacy of the liquid biopsy as a source of biomarkers for detecting UTUC. Using the third-generation high-definition single-cell assay (HDSCA3.0) workflow, we investigate liquid biopsy samples collected from patients with UTUC and normal donors (NDs) to provide critical information regarding the molecular and morphological characteristics of circulating rare events. We document several important findings from the liquid biopsy analysis of patients diagnosed with UTUC prior to surgery: (1) Large extracellular vesicles (LEVs) and circulating tumor cells (CTCs) are detectable in the peripheral blood. (2) The rare-event profile is highly heterogeneous. (3) Clinical data elements correlate with liquid biopsy analytes. Overall, this study provides evidence for the efficacy of the liquid biopsy in understanding the biology of UTUC with the future intent of informing clinical decision making, ultimately improving patient outcomes.

## 1. Introduction

Urothelial carcinomas (UCs) are a broad and heterogeneous group of malignancies that are among the most frequently occurring tumor indications in Europe and the United States [1]. Within these, the prevalence of upper tract urothelial carcinoma (UTUC) is rare, accounting for only 5–10% of total malignancies and an annual incidence of 2 cases per 100,000 individuals [2]. UTUCs are classified as non-muscle-invasive (Tis, Ta, T1) or muscle-invasive (T2-T4), indicative of tumor invasion into the muscularis, peripelvic fat, renal parenchyma, periureteric fat, and/or other organs [3]. Approximately 50–60% of cases present as muscle-invasive at diagnosis [4]. While low-grade tumors are unlikely to spread from the kidney or ureter, high-grade UTUCs have a higher potential to recur elsewhere in the body. Bladder recurrence occurs in 22–47% of patients, while approximately 17% of patients manifest concomitant bladder cancer [5,6].

The current standard tools for the diagnosis and preoperative risk stratification of primary UTUC include urine cytology, cross-sectional imaging, and ureteroscopic (URS) biopsy, the procedure that informs the need for kidney-sparing surgery (KSS) for low-risk disease and radical nephroureterectomy (RNU) for high-risk disease [7]. While minimally invasive, URS biopsy leaves more to be desired in the accurate matching of biopsy grade and final pathology (66% in low-grade and 97% in high-grade tumors) [8]. Additionally, under-grading and under-staging rates from URS biopsy remain a concern, at 32% and 46%, respectively [8]. Furthermore, analyses show that, for patients with UTUC who undergo RNU, concordance between URS biopsy clinical staging and RNU pathological staging was found to be 34.5%, underscoring the significant discrepancy between clinical stage and final pathology [9]. Moreover, URS biopsy is associated with a higher rate of intravesical recurrence following RNU [10,11]. Taken together, such evidence necessitates the need for additional toolsets to assist the current clinical paradigm of UTUC patient care. Herein, we discuss the efficacy of the liquid biopsy as a source of biomarkers for detecting UTUC and informing treatment options to improve patient outcomes.

The detection and enumeration of circulating tumor cells (CTCs) via the liquid biopsy, typically accomplished through the collection of peripheral blood (PB), are deemed viable prognostic markers for relapse [12,13,14]. A study by Claps et al. illustrated that a downward trend of CTCs present in patient liquid biopsy was indicative of progression-free survival and increased overall survival [15]. Similarly, a meta-analysis by Zhang et al. investigating the relationship between UTUC and the presence of CTCs in the liquid biopsy showed that the presence of CTCs was indicative of negative patient outcomes with regard to tumor staging and metastasis [14]. These studies support the assertion that CTCs found in PB are associated with poorer patient prognosis. The current study will make use of the third-generation liquid biopsy workflow for the systematic visualization and characterization of CTCs and large extracellular vesicles (LEVs) present in the PB collected from patients newly diagnosed with primary UTUC prior to surgical resection.

The third-generation high-definition single-cell assay (HDSCA3.0) workflow provides critical information regarding the molecular and morphological characteristics of rare events detected in the liquid biopsy with immunocytochemistry [16] coupled with downstream molecular characterization to deliver diagnostic pathology-quality data for clinical decision making [17,18,19,20,21,22]. Through enumeration, events in a sample, primarily CTCs and LEVs, can be discriminated and then quantified based on size, morphology, DNA load, and phenotype [23], providing a comprehensive summary of the disease state. The primary objective of this study was to investigate the prognostic relationship between CTCs and disease state in the PB samples collected from patients diagnosed with UTUC taken prior to RNU. In the assessment of this relationship, the presence of CTCs in reference to clinical data metrics will also be examined, namely, clinical and pathological staging as well as histological subtype. Overall, this study aims to provide evidence for the efficacy of the liquid biopsy in further understanding UTUC with the future intent to predict metastatic relapse post-surgery, ultimately improving patient outcomes.

## 2. Materials and Methods

### 2.1. Study Design

This was a single institution prospective study of patients diagnosed with primary UTUC in which PB samples were collected prior to surgery. Eligible patients underwent surgical removal of the primary tumor with curative intent. Samples were collected from the University of Southern California’s Keck School of Medicine between May 2021 and March 2022. Prospectively collected clinical, radiologic, and pathologic data elements, as well as a limited amount of follow-up data, were also provided. Recurrence was defined as any clinical recurrence, the majority shown radiologically, either symptomatic or not. Patient recruitment took place according to institutional review board’s approved protocols (IRB no. HS-19-00886 (liquid biopsy collection) and no. HS-17-00168 (clinical and demographic data collection)), and all study participants provided written informed consent. Here, we present the liquid biopsy analysis from a total of 20 patients diagnosed with UTUC. Additionally, 50 normal donor (ND) blood samples from individuals with no known pathology were procured from Epic Sciences (San Diego, CA, USA).

### 2.2. Blood Sample Processing

As previously described, PB blood samples were collected in 10 mL blood collection tubes (Cell-free DNA BCT, Streck, La Vista, NE, USA) within 24–48 h prior to processing by the Convergent Science Institute in Cancer (CSI-Cancer) at the University of Southern California [16]. Samples then underwent brief red blood cell lysis in isotonic ammonium chloride solution, after which the entire nucleated cell fraction was plated on custom glass slides (Marienfeld, Lauda, Baden-Württemberg, Germany) at approximately 3 million cells per slide. Slides were blocked with 7% BSA and dried prior to cryostorage at −80 °C and subsequent rare-event analysis.

Negative and positive controls were incorporated as contrived samples. The negative control was the ND blood, and the positive control was ND blood spiked with cell lines with a known expression profile, as previously described [16]. These controls were processed according to standard operating procedures and were used throughout the HDSCA workflow.

### 2.3. Blood Sample Staining and Imaging

For rare-event analysis, each test included two slides per sample. In this study, there was an average of 1.3 mL of blood analyzed per test. Slides were stained at room temperature using the IntelliPATH FLXTM autostainer (Biocare Medical LLC, Irvine, CA, USA), as previously described [24,25], with 2.5 ug/mL of a mouse IgG1 anti-human CD31:Alexa Fluor^®^ 647 mAb (clone: WM59, MCA1738A647, BioRad, Hercules, CA, USA) and 100 ug/mL of goat anti-mouse IgG monoclonal Fab fragments (115–007- 003, Jackson ImmunoResearch, West Grove, PA, USA), permeabilized using 100% cold methanol, followed by an antibody cocktail consisting of mouse IgG1/Ig2a anti-human cytokeratins (CKs) 1, 4, 5, 6, 8, 10, 13, 18, and 19 (clones: C-11, PCK-26, CY-90, KS-1A3, M20, A53-B/A2, C2562, Sigma, St. Louis, MO, USA), mouse IgG1 anti-human CK 19 (clone: RCK108, GA61561–2, Dako, Carpinteria, CA, USA), mouse anti-human CD45:Alexa Fluor^®^ 647 (clone: F10–89-4, MCA87A647, AbD Serotec, Raleigh, NC, USA), and rabbit IgG anti-human vimentin (Vim) (clone: D21H3, 9854BC, Cell Signaling, Danvers, MA, USA). Lastly, slides were incubated with Alexa Fluor^®^ 555 goat anti-mouse IgG1 antibody (A21127, Invitrogen, Carlsbad, CA, USA) and 4′,6-diamidino-2-phenylindole (DAPI; D1306, ThermoFisher, Waltham, MA, USA) prior to being mounted with a glycerol-based aqueous medium. Automated high-throughput fluorescence scanning microscopy at 10× objective magnification was then used to obtain 2304 frame images per fluorescence channel per side.

### 2.4. Rare-Event Detection and Classification

As previously reported [24,25], the detection and classification of rare events employed OCULAR (Outlier Clustering Unsupervised Learning Automated Report), a custom computational methodology designed to detect rare cell and LEV candidates [23]. A total of 761 morphometric parameters were provided for each event based on nuclear and cytoplasmic morphology, as well as biomarker expression (CK, Vim, CD45/CD31) from the 4-channel immunofluorescence assay (DAPI, AlexaFluor^®^ 488, AlexaFluor^®^ 555, AlexaFluor^®^ 647).

Manual reporting was completed on computationally identified events to confirm signal intensity and distribution, as well as morphology. Images of candidate rare events were presented to a hematopathologist-trained technical analyst for analysis and interpretation, in which rare events were classified into 12 categories (8 cellular, 4 LEV) based on the 4 fluorescent channels, creating a combination of biomarker expressions. Cells defined by a distinctly appearing nucleus by DAPI morphology presenting as CK-positive, Vim-negative, and CD45/CD31-negative were classified as epithelial-like CTCs (epi.CTCs). Epi.CTCs expressing Vim were further specified as mesenchymal-like CTCs (mes.CTCs). To contextualize the number of rare events identified within PB samples, automatically determined white blood cell (WBC) counts (Medonic M-series Hematology Analyzer, Clinical Diagnostic Solutions Inc., Fort Lauderdale, FL, USA) were used in conjunction with the number of WBCs detected by the assay per slide to calculate the amount of blood analyzed per test. As such, results of rare-event detection and classification are presented as fractional values of events/mL.

The identification of LEVs closely followed the OCULAR methodology outlined above; candidates were CK-positive with variable Vim and CD45/CD31 expression. Additional consideration was given in manually classifying LEV candidates as either free-floating or in close proximity to other cells. Further corrections during the manual classification of LEVs included the exclusion of bubbles, halos, or light refractions resembling the round and membranous morphology of LEVs in the examination of CK imaging frames. A maximum threshold of three LEVs per frame was used to exclude CK-positive debris that may have been introduced onto the slide during processing and/or staining.

### 2.5. Statistical Analysis

To calculate the correlations between clinical and demographic data and the rare events detected in the liquid biopsy samples, the Spearman’s rank correlation [26] and Mann–Whitney U test were applied, also known as the Wilcoxon rank-sum test [27,28]. For continuous clinical variables and variables with ordinal encoding, the Spearman’s rank correlation was used. Furthermore, for all categorical variables, the Wilcoxon rank-sum test was used for every variable pair. Statistical significance was determined at a *p*-value ≤ 0.05. The two statistical tests used were non-parametric tests, which matched the conditions of the variables used. The analysis performed herein is similar to a sister study of bladder cancer patients [25]. All computational analyses and visualizations were performed in python (version 3.8.5, https://www.python.org/, accessed on 11 May 2022) utilizing the scipy (version 1.5.0, https://scipy.org/, accessed on 11 May 2022), scikit-learn (version 0.23.2, https://scikit-learn.org/stable/, accessed on 11 May 2022), and matplotlib (version 3.2.2, https://matplotlib.org/, accessed on 11 May 2022) packages.

For visualizing the variation of morphological parameters of detected cellular events and to uncover their variation within the channel-based classification, a two-dimensional tSNE (t-distributed stochastic neighbor embedding) was used [29]. Along with the tSNE, a clustering algorithm was used to identify cellular subgroups beyond the channel-type classifications. An agglomerative clustering algorithm, imported from the sklearn library version 0.23.2, https://scikit-learn.org/stable/ (accessed on 11 May 2022) [30], was applied utilizing Ward’s linkage and a Euclidean distance metric [31].

Negative and positive controls were incorporated as contrived samples. The negative control was ND blood, and the positive control was the normal blood donor spiked with cell lines with a known expression profile (i.e., SKBR3 or HPAEC). These controls were processed according to standard operating procedures and included in the immunofluorescence staining, scanning, and rare-event detection. 

## 3. Results

A total of 20 patients with primary UTUC were accrued for this study, each providing a single PB sample obtained prior to surgery. Demographic and clinical data metrics were collected and are provided in Table 1. The demographic information for the NDs was limited to the individuals’ ages (median 57; range 45–82; mean 58.9 years).

### 3.1. Liquid Biopsy Analysis Prior to Surgery

The complete blood cell count was measured prior to blood processing at CSI-Cancer. For all samples in this study, the average cell count per frame was 1430.80 ± 343.85 (UTUC: 1326.20 ± 344.42; ND: 1472.64 ± 343.62). For the 20 UTUC samples included here, there was a median WBC count of 6.65 (range 2.8–9.8; mean 6.01) million cells/mL PB. Across all UTUC samples, total rare-event detection had a median of 178.23 events/mL (range 25.66–779.80; mean 234.88). For ND samples, total rare-event detection had a median of 38.50 events/mL (range 4.39–141.55; mean 47.86). A significant difference in the total rare-event (i.e., cells and LEVs) count/mL was observed between the patients diagnosed with UTUC and the NDs (*p*-value < 0.0001).

### 3.2. Rare-Cell Characterization

**Figure 1:**
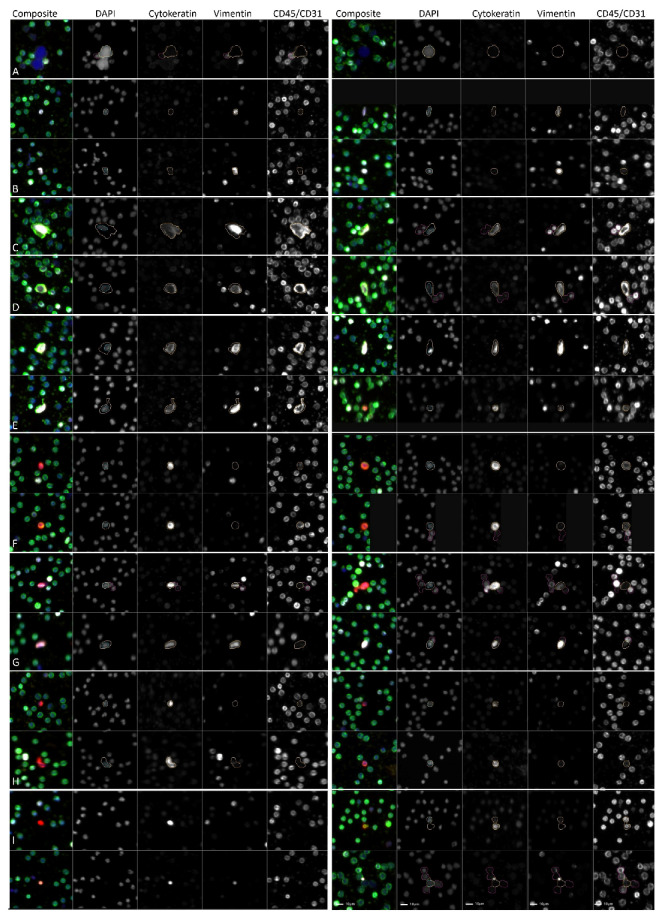
Gallery of representative rare events detected in PB samples collected from patients diagnosed with UTUC (1326.20 ± 344.42 cells/frame) prior to surgery or NDs (1472.64 ± 343.62 cells/frame) with no known pathology by HDSCA3.0. (**A**–**H**) rare cells and (**I**) LEVs. (**A**) DAPI only; (**B**) Vim; (**C**) CD45/CD31; (**D**) Vim|CD45/CD31; (**E**) CK|Vim|CD45/CD31; (**F**) CK|CD45/CD31; (**G**) mes.CTC; (**H**) epi.CTC; (**I**) LEVs (top left: CK only; bottom left: CK|Vim|CD45/CD31; top right: CK|CD45/CD31; bottom right: CK|Vim.) Blue: DAPI, Red: CK, White: Vim, Green: CD45/CD31. Images taken at 100× magnification. Scale bar = 10 µm.

**Figure 2:**
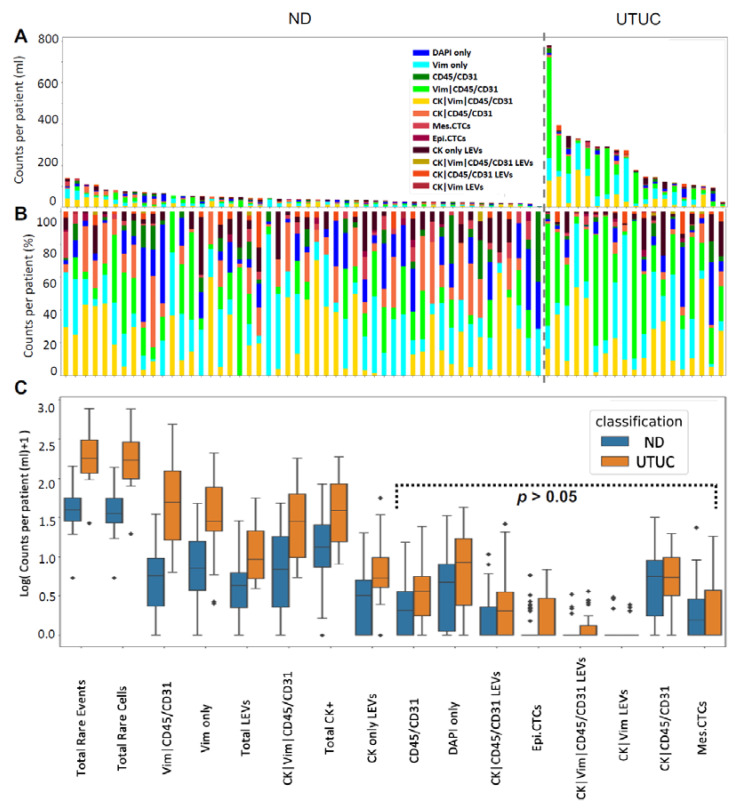
Rare-event detection in PB samples collected from UTUC patients prior to surgery using HDSCA3.0. (**A**) Enumeration and (**B**) frequency of each rare event by channel-type classification. (**C**) Graphical representation of the channel-type rare events/mL ordered by degree of statistical significance between UTUC and ND samples. Channel-type specifications that were not statistically significant across ND and UTUC samples are highlighted (*p*-value > 0.05). Diamond points indicate outliers.

We identified eight cellular categories that were defined by a nuclear DAPI signal and depended on the different biomarker expression profiles. Figure 1 shows a gallery of CTCs, while the graphical representations of the enumeration and frequency of each rare event identified per test for each patient sample are shown in Figure 2, respectively. Total rare-cell detection for the UTUC samples had a median of 169.85 cells/mL (range 18.62–771.63; mean 219.83). The ND samples presented with a median rare-cell detection of 34.46 cells/mL (range 4.39–137.03; mean 43.21). A statistically significant difference in total rare-cell detection was observed between the UTUC patients and ND samples (*p*-value < 0.0001).

Total CK-positive cells were detected with a median of 37.91 events/mL (range 7.06–186.59; mean 60.37) from all UTUC samples. The ND samples had a median of 12.40 cells/mL (range 0–83.24; mean 18.96). A statistically significant difference in total CK-positive cell detection was observed between the UTUC and ND samples (*p*-value = 0.0015). All patient samples had CK-positive cells detected. Within the total rare-cell population, the frequency of CK-positive cells detected varied. Overall, there was a median frequency of 18.84% (range 3.98–74.17%; mean 28.89%) in the UTUC samples.

Epi.CTCs were detected with a median of 0 cells/mL (range 0–5.90; mean 1.30), while mes.CTCs were detected with a median of 0 cells/mL (range 0–17.00; mean 2.24) in the UTUC samples. There was no statistically significant difference between the UTUC and ND samples in epi.CTCs/mL or mes.CTCs/mL observed. Eleven patients (55%) did not present with epi.CTCs or with mes.CTCs. Only seven (35%) patients overlapped in presenting with 0 cells/mL for both epi.CTCs and mes.CTCs.

Additional candidate CTCs detected in UTUC patient samples included CK|Vim|CD45/CD31 (median 27.30 cells/mL; range 4.47–179.08; mean 51.10) and CK|CD45/CD31 (median 4.54 cells/mL; range 0–18.79; mean 5.73). Other detectable rare cells included morphologically distinct Vim|CD45/CD31 (median 48.40 cells/mL; range 5.39–485.75; mean 89.47), Vim only (median 27.54 cells/mL; range 1.56–207.76; mean 54.50), CD45/CD31 only (median 2.68 cells/mL; range 0–23.27; mean 4.37), and DAPI only cells (median 7.58 cells/mL; range 0–41.70; mean 11.12). The UTUC samples had a higher cellular enumeration of Vim only and Vim|CD45/CD31 cells (*p*-value < 0.0001, for both; Figure 2C). The biological significance of these cellular populations has yet to be determined. Enumerations of all rare events in both UTUC and ND samples can be found in Supplementary Table S1.

The most prevalent cell types detected in the PB of patients with UTUC were Vim|CD45/CD31 (median 28.57%; range 2.41–88.06%; mean 35.17%), Vim only (median 19.23%; range 2.22–82.53%; mean 24.27%), CK|Vim|CD45/CD31 (median 14.10%; range 2.17–61.67%; mean 23.46%), and lastly, DAPI only (median 5.66%; range 0–46.67%; mean 9.01%). Out of all the rare cells observed across patient samples, Vim|CD45/CD31 cells constituted 44.83%, Vim only cells 20.33%, CK|Vim|CD45/CD31 24.55%, and DAPI only cells constituted 4.16%. A positive correlation was identified between DAPI only and CD45/CD31 only cells (Spearman coefficient = 0.62, *p*-value = 0.0047), mes.CTC and Vim only cells (Spearman coefficient = 0.57, *p*-value = 0.01), Vim|CD45/CD31 and CD45/CD31 only cells (Spearman coefficient = 0.49, *p*-value = 0.03), mes.CTC and CK|Vim|CD45/CD31 cells (Spearman coefficient = 0.47, *p*-value = 0.04), and CK|CD45/CD31 and CK|Vim|CD45/CD31 cells (Spearman coefficient = 0.47, *p*-value = 0.04). This suggests that the cellular populations defined by channel-type classification are associated with one another and represent the heterogeneity of UTUC.

**Figure 3:**
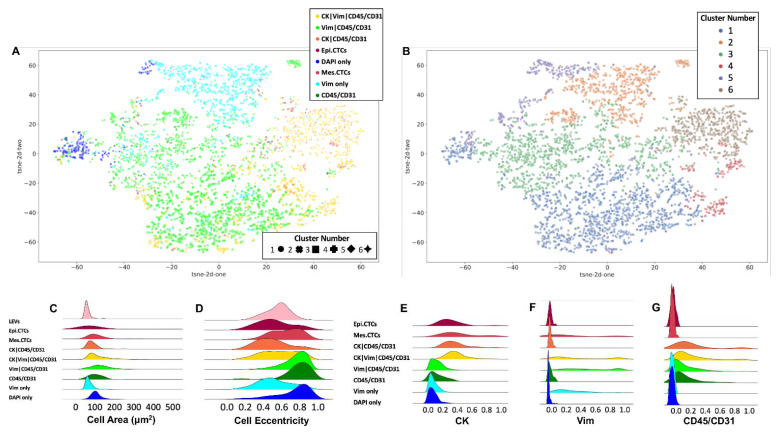
Morphometric analysis of individual events detected by HDSCA3.0 in PB samples collected from patients with UTUC prior to RNU. Depicted is a tSNE plot of rare cellular events depicting the underlying morphological heterogeneity. Each point represents a single cell and is color-coded according to (**A**) its channel-type classification and (**B**) a distinct cluster number, as determined by a clustering algorithm. Also visualized are the probability density distributions for select morphometric parameters across channel-type classifications of (**C**) cell area, (**D**) cell eccentricity, (**E**) median CK signal intensity, (**F**) median Vim signal intensity, and (**G**) median CD45/CD31 signal intensity.

The cellular morphometrics were used to further investigate the heterogeneity of the rare-cell populations detected in the PB of patients with UTUC. A dimensionality reduction algorithm and a clustering algorithm were applied to visualize the cellular heterogeneity in a two-dimensional plane. The morphometrics considered included the area and the eccentricity of the cells and the nucleus, respectively. Furthermore, the median intensity of the four channels was also used as input. The resulting cellular tSNE, depicted in Figure 3A,B, is color-coded according to channel-type classification and cluster number, respectively. Cellular area and eccentricity, along with the median channel intensities, are plotted in Figure 3C–G. For example, the cellular heterogeneity within classifications is evident for mes.CTCs, with the appearance of two peaks in the distributions for cell eccentricity, median CK, and Vim expression.

The tSNE depicted in Figure 3 is a visualization of the morphological relationships between channel classifications, with each point depicting a single cellular event. The cells split into clusters, with morphological heterogeneity driving separation within channel classifications. For instance, the cells in the Vim|CD45/CD31 classification are divided between clusters 1, 2, 3, and 5. Cluster 1 contains the majority of Vim|CD45/CD31 cells, along with CK|Vim|CD45/CD31 cells and DAPI only cells. Cluster 2 includes the majority of Vim only cells and a subset of Vim|CD45/CD31 cells. In addition, cluster 3 contains a mixture of Vim only, DAPI only, and Vim|CD45/CD31 cells. Cluster 5 has cells across the DAPI only, Vim only, and Vim|CD45/CD31 categories. Finally, clusters 4 and 6 contain the remaining CK-positive cells.

### 3.3. LEV Detection

LEVs were classified by a lack of DAPI signal and positive CK signal, as well as morphology. Total LEV detection for the UTUC samples was a median of 8.33 LEVs/mL (range 2.97–54.94; mean 15.05; Figure 2). The ND samples presented with a median of 3.34 LEVs/mL (range 0–27.91; mean 4.65). There was a significant difference in LEVs/mL detected between the ND and UTUC samples (*p*-value < 0.0001). In the UTUC samples, LEVs were detected either alone (*n* = 118; 50.9%) or in close proximity to cells (*n* = 114; 49.1%). In the ND samples, these LEV populations totaled 85 (45.9%) and 100 (54.1%), respectively.

CK only LEVs were detected in 19 UTUC samples (95%), with a median of 4.34 LEVs/mL (range 0–54.94; mean 10.36). CK|CD45/CD31 LEVs were also detected in 11 patients (55%), with a cohort median of 1.04 LEVs/mL (range 0–25.08; mean 4.05). The most infrequent LEVs detected were CK|Vim|CD45/CD31 (median 0 LEVs/mL; range 0–2.64; mean 0.44) and CK|Vim (median 0 LEVs/mL; range 0–1.47; mean 0.20), in 25% and 15% of patients, respectively. A negative correlation was observed between CK only LEVs and CK|Vim|CD45/CD31 LEVs (Spearman coefficient = −0.54, *p*-value = 0.02) and between CK|Vim|CD45/CD31 LEVs and epi.CTCs (Spearman coefficient = −0.48, *p*-value = 0.04). The CK only LEV population was observed at a significantly higher level in the UTUC samples than in the ND samples (*p*-value = 0.002). These data suggest that LEVs represent a new potential analyte of tumor heterogeneity with the potential to monitor disease status.

### 3.4. Correlation with Clinical Data

Correlation analysis was used to determine the relationship between the clinical and demographic metrics collected for the patients with UTUC (*n* = 20) and the various liquid biopsy analytes detected. Here, we report only the significant correlations; for variables analyzed under the Wilcoxon signed-rank test, only those having four or more patients were reported. A complete table of all comparisons can be found in Supplementary File S1. A negative correlation was detected between height and weight and mes.CTCs/mL (Spearman coefficient = −0.59 and −0.57, respectively; *p*-value = 0.01 for both), as well as preoperative glomerular filtration rate (GFR) and CK|CD45/CD31 cells (Spearman coefficient = −0.55, *p*-value = 0.01). A positive correlation was found between age and CK|Vim|CD45/CD31 cells, total CK-positive events, and Vim only cells (Spearman coefficient = 0.57, 0.56, and 0.48, respectively; *p*-value = 0.01, 0.01, and 0.04, respectively). The total Charlson Comorbidity Index (CCI) score for all comorbidities analyzed correlated with CK|Vim|CD45/CD31 cells, CK|CD45/CD31 cells, total CK-positive cells, and Vim only cells (Spearman coefficient = 0.56, 0.56, 0.55, and 0.53, respectively; *p*-value = 0.01, 0.01, 0.01, and 0.02, respectively). CCI status (i.e., 0 vs. ≥1) correlated with CK|Vim|CD45/CD31, total CK-positive, Vim only, and CK|CD45/CD31 cells/mL (Wilcoxon = −2.45, −2.37, −2.37, and −2.23, respectively; *p*-value = 0.01, 0.02, 0.02, 0.02, respectively). Patient ASA (American Society of Anesthesiologists) scores positively correlated with Vim|CD45/CD31 cells/mL (Wilcoxon = 2.02, *p*-value = 0.04). Where the tumor was located, specifically in the pelvic/renal region and distal ureter, was found to positively correlate with Vim only cells (Wilcoxon = 2.18, *p*-value = 0.03). Finally, a negative correlation was identified between patients having previous bladder cancer and CK only LEVs/mL (Wilcoxon = −2.13, *p*-value = 0.03).

For the 20 patients diagnosed with UTUC, recurrence data post-surgery were collected. However, the majority of the patients had short follow-up times, with a median follow-up of 111.5 days after surgery. Thus, to perform correlation analysis, the follow-up time after surgery was taken into account. To that end, we considered patients with recurrence and follow-up information of 6 months and 130 days, which limited the dataset to six and five patients, respectively. Within the two timeframes, three patients recurred within 6 months, and two patients recurred within 5 months. No statistically significant differences were found when performing the correlation analysis for recurrence. Additional time is needed for the data to mature in order to elucidate further correlations between recurrence status and liquid biopsy analytes.

## 4. Discussion

This study shows the detection of liquid biopsy analytes that allow for the stratification of NDs from patients diagnosed with UTUC prior to RNU. Combined with current clinical standards, the liquid biopsy has the potential to provide diagnostic confidence or prognostic utility around treatment response and the monitoring of minimal residual disease. We document several important findings from the liquid biopsy analysis of patients diagnosed with UTUC prior to surgery: (1) LEVs and CTCs are detectable in the PB. (2) The rare-event profile is highly heterogeneous. (3) Clinical data elements correlate with liquid biopsy analytes. As a non-invasive tool for the discovery of cancer-related biomarkers, the liquid biopsy has the potential to represent the complex process of tumorigenesis and metastasis. The data presented here suggest that liquid biopsy analysis for CTCs and LEVs should be further investigated in a larger cohort for inclusion in UTUC patient care.

Total rare cells and LEVs were shown to be detected in UTUC samples at a significantly higher count than in ND samples (median 169.85 cells/mL vs. 34.46 cells/mL and 8.33 LEVs/mL vs. 3.34 LEVs/mL, respectively). Additionally, both positive and negative correlations were identified between multiple liquid biopsy analytes and clinical data metrics, including CCI status, tumor location, and previous bladder cancer status. A promising new analyte for UTUC patient care detected in an enrichment-free liquid biopsy approach are circulating LEVs. The four different LEV classifications detected in this study represent further tumor heterogeneity and were demonstrated to be present at significantly higher counts in patients with UTUC prior to RNU compared to the NDs. The presence of tumor-associated LEVs is most likely the result of the presence of an intact primary tumor in the patient. Exosomes contain various analytes (RNA, DNA, proteins, and metabolites) concordant with the parental cell properties, suggesting they are a promising alternative to circulating tumor DNA (ctDNA) or CTCs as disease biomarkers.

The liquid biopsy may be a source for detecting biomarkers of micrometastases indicative of early disease dissemination, and the assessment of these data prior to surgical intervention is critical. A meta-analysis of 2161 UC patients indicated that the presence of CTCs in the PB correlated with histological grade, tumor stage, and metastasis while being an independent predictive indicator of poor outcomes [14]. It is important to note that this analysis included 30 different published studies using various methods for CTC detection. Therefore, patients with low CTC counts prior to undergoing RNU are hypothesized to have a lower chance of recurrence and are therefore encouraged to receive surgical treatment. Using the HDSCA3.0 workflow, we detected both epi.CTCs and mes.CTCs in a subset of patient samples (45%), but no statistical difference was found compared to ND samples for these cell frequencies. Despite this, additional candidate CTCs were observed at higher frequencies, including CK|Vim|CD45/CD31 (median 27.30 cells/mL) and CK|CD45/CD31 (median 4.54 cells/mL). In metastatic prostate cancer, the rare-cell populations expressing CK|CD45/CD31 included platelet-coated epi.CTCs, which was determined to be a predictive biomarker for treatment response [24]. Understanding the molecular role of platelets in CTC biology will allow for the identification and characterization of clinically relevant CTCs for patient care [32]. Foerster et al. show that, in a large cohort of patients diagnosed with UTUC, mean platelet counts increased with advanced stage and tumor size [33]. Platelets may be indicative of more aggressive disease, and further proteomic characterization is warranted to understand the biological significance of this cellular population. This study highlights the promise of the liquid biopsy for the early risk stratification of patients with UTUC, prediction of disease outcome, and early detection of metastatic relapse.

Our analysis revealed a heterogeneous rare-cell population in the PB of patients with UTUC. Eight unique rare-cell categories were defined by their expression of CK, Vim, and CD45/CD31. Each cellular category included a mixture of cell types, which can be exemplified through the morphometric parameters, such as cellular eccentricity and size. The CCI status negatively correlated with various liquid biopsy rare-cell types; therefore, we conclude that the rare cells detected are representative of a range of comorbid conditions. Furthermore, the detection of Vim only cells in the PB correlated with the primary tumor location. This is evidence for the circulation of various rare cells as a measure of overall disease state. Further molecular analysis on the rare cells detected is needed and could help sub-stratify cells within each channel-type classification. For example, single-cell RNA sequencing could reveal the origin of the Vim only cells to further understand the association with specific tumor locations [34]. Here, we show that circulating rare cells are associated with a disease state and may be critical towards understanding a more comprehensive liquid biopsy profile beyond CTCs.

Molecular characterization of both circulating rare cells and LEVs detected in this study will elucidate their role in the processes associated with UTUC tumorigenesis and metastasis. Understanding the genomic architecture and proteome will reveal elements attributing to tumor growth, treatment resistance, and metastasis to inform clinical decision making for patient-centered treatment and the development of novel therapies. The HDSCA3.0 workflow provides the opportunity for a comprehensive liquid biopsy analysis via targeted multiplexed proteomics and single-cell genomic analysis [17,18,20,22,23,35], as well as ctDNA analysis [36,37,38]. Prior studies indicated that high ctDNA levels were associated with progression and metastatic disease [39,40,41]. With the use of a single platform, it is possible to characterize tumor heterogeneity through the combination of methodologies to provide a precision medicine approach for patients diagnosed with UTUC. Future research will focus on establishing evidence for the clinical utility of the liquid biopsy in UTUC to predict relapse post-RNU and to assist in clinical decision making to improve patient outcomes.

## 5. Conclusions

This study demonstrates initial evidence for the clinical utility of the liquid biopsy in UTUC patient care with the future objective of predicting recurrence post-surgery, enabling clinical intervention(s) that can improve patient outcomes. Here, we find that rare cells and LEVs can be used to distinguish patients with UTUC from NDs, with distinct populations indicating inter- and intra-patient heterogeneity through the liquid biopsy. While additional studies are warranted to uncover the power of liquid biopsy analytes to predict recurrence, the findings from this study support the liquid biopsy as a promising clinical tool for UTUC patient care.

## Figures and Tables

**Table 1:** Clinical demographics for UTUC patients. CCI = Charlson Comorbidity Index.

Variable	Category	Value
Age	Median (range), year	66.5 (43–88)
BMI	Median (range), kg/m^2^	26.05 (17.6–37.8)
Gender	Male	17 (85%)
	Female	3 (15%)
Smoker	Never	8 (40%)
	Former	8 (40%)
	Current	4 (20%)
CCI	0	10 (50%)
	≥1	10 (50%)
Previous Bladder Cancer	Yes	15 (75%)
	No	5 (25%)
Type of Surgery	Nephroureterectomy +/− Bladder Cuff Excision	17 (85%)
	Distal Ureterectomy	2 (10%)
	Laser Ablation	1 (5%)
Histology	Pure Urothelial	17 (85%)
	Urothelial with Variant Histology	2 (10%)
	N/A *	1 (5%)
Pathology Grade	Low	3 (15%)
	High	16 (80%)
	N/A *	1 (5%)
RNU pTstage	pT0	0 (0%)
	pTa	10 (50%)
	pTis	0 (0%)
	pT1	1 (5%)
	pT2	0 (0%)
	pT3	7 (35%)
	pT4	1 (5%)
	N/A *	1 (5%)
Lymph Node Status	Node+	16 (80%)
	Node−	3 (15%)
	N/A *	1 (5%)
Neoadjuvant Chemo	Yes	7 (35%)
	No	13 (65%)
Adjuvant Chemo	Yes	1 (5%)
	No	19 (95%)
Recurrence (Bladder)	Yes	3 (15%)
	No	17 (85%)

* N/A values correspond to a patient that received Laser Ablation, and thus, there was no gross pathology specimen.

## Data Availability

All data discussed in this manuscript are included in the main manuscript text or supplementary materials. The imaging data are available through the BloodPAC Data Commons, Accession ID “BPDC000125” (https://data.bloodpac.org/discovery/BPDC000125).

## Supplementary Materials

The following are available online at https://www.mdpi.com/article/10.3390/cancers14123007/s1, File S1: Excel file for correlation analysis; Table S1: Enumeration/mL of rare events by channel-type classification ordered by degree of statistical significance between UTUC and ND samples.

## References

[1] Bersanelli M., Buti S., Giannatempo P., Raggi D., Necchi A., Leonetti A., Banna G.L., Petrelli F. (2021). Outcome of patients with advanced upper tract urothelial carcinoma treated with immune checkpoint inhibitors: A systematic review and meta-analysis. Crit. Rev. Oncol..

[2] Rouprêt M., Babjuk M., Burger M., Capoun O., Cohen D., Compérat E.M., Cowan N.C., Dominguez-Escrig J.L., Gontero P., Mostafid A.H. (2021). European Association of Urology Guidelines on Upper Urinary Tract Urothelial Carcinoma: 2020 Update. Eur. Urol..

[3] Honda Y., Nakamura Y., Teishima J., Goto K., Higaki T., Narita K., Akagi M., Terada H., Kaichi Y., Fujii S. (2019). Clinical staging of upper urinary tract urothelial carcinoma for T staging: Review and pictorial essay. Int. J. Urol..

[4] Petros F.G. (2020). Epidemiology, clinical presentation, and evaluation of upper-tract urothelial carcinoma. Transl. Androl. Urol..

[5] Xylinas E., Rink M., Margulis V., Karakiewicz P., Novara G., Shariat S.F. (2012). Multifocal carcinoma in situ of the upper tract is associated with high risk of bladder cancer recurrence. Eur. Urol..

[6] Cosentino M., Palou J., Gaya J.M., Breda A., Rodriguez-Faba O., Villavicencio-Mavrich H. (2013). Upper urinary tract urothelial cell carcinoma: Location as a predictive factor for concomitant bladder carcinoma. World J. Urol..

[7] Leow J., Liu Z., Tan T.W., Lee Y.M., Yeo E.K., Chong Y.-L. (2020). Optimal Management of Upper Tract Urothelial Carcinoma: Current Perspectives. Onco Targets Ther..

[8] Subiela J.D., Territo A., Mercadé A., Balañà J., Aumatell J., Calderon J., Gallioli A., González-Padilla D.A., Gaya J.M., Palou J. (2020). Diagnostic accuracy of ureteroscopic biopsy in predicting stage and grade at final pathology in upper tract urothelial carcinoma: Systematic review and meta-analysis. Eur. J. Surg. Oncol..

[9] Mori K., Katayama S., Laukhtina E., Schuettfort V.M., Pradere B., Quhal F., Motlagh R.S., Mostafaei H., Grossmann N.C., Rajwa P. (2022). Discordance between Clinical and Pathological Staging and Grading in Upper Tract Urothelial Carcinoma. Clin. Genitourin. Cancer.

[10] Marchioni M., Primiceri G., Cindolo L., Hampton L.J., Grob M.B., Guruli G., Schips L., Shariat S.F., Autorino R. (2017). Impact of diagnostic ureteroscopy on intravesical recurrence in patients undergoing radical nephroureterectomy for upper tract urothelial cancer: A systematic review and meta-analysis. BJU Int..

[11] Katims A.B., Say R., Derweesh I., Uzzo R., Minervini A., Wu Z., Abdollah F., Sundaram C., Ferro M., Rha K. (2021). Risk Factors for Intravesical Recurrence after Minimally Invasive Nephroureterectomy for Upper Tract Urothelial Cancer (ROBUUST Collaboration). J. Urol..

[12] Fu G., Cheng K.S., Chen A., Xu Z., Chen X., Tian J., Xu C., Sun Y., Neoh K.H., Dai Y. (2021). Microfluidic Assaying of Circulating Tumor Cells and Its Application in Risk Stratification of Urothelial Bladder Cancer. Front. Oncol..

[13] Soave A., Riethdorf S., Dahlem R., Minner S., Weisbach L., Engel O., Fisch M., Pantel K., Rink M. (2017). Detection and oncological effect of circulating tumour cells in patients with variant urothelial carcinoma histology treated with radical cystectomy. BJU Int..

[14] Zhang Z., Fan W., Deng Q., Tang S., Wang P., Xu P., Wang J., Yu M. (2017). The prognostic and diagnostic value of circulating tumor cells in bladder cancer and upper tract urothelial carcinoma: A meta-analysis of 30 published studies. Oncotarget.

[15] Claps F., Mir M.C., Zargar H. (2021). Molecular markers of systemic therapy response in urothelial carcinoma. Asian J. Urol..

[16] Marrinucci D., Bethel K., Kolatkar A., Luttgen M.S., Malchiodi M., Baehring F., Voigt K., Lazar D., Nieva J.J., Bazhenova L. (2012). Fluid biopsy in patients with metastatic prostate, pancreatic and breast cancers. Phys. Biol..

[17] Gerdtsson A.S., Thiele J.-A., Shishido S.N., Zheng S., Schaffer R., Bethel K., Curley S., Lenz H.-J., Hanna D.L., Nieva J. (2019). Single cell correlation analysis of liquid and solid biopsies in metastatic colorectal cancer. Oncotarget.

[18] Malihi P.D., Graf R.P., Rodriguez A., Ramesh N., Lee J., Sutton R., Jiles R., Velasco C.R., Sei E., Kolatkar A. (2020). Single-Cell Circulating Tumor Cell Analysis Reveals Genomic Instability as a Distinctive Feature of Aggressive Prostate Cancer. Clin. Cancer Res..

[19] Thiele J.A., Pitule P., Hicks J., Kuhn P. (2019). Single-Cell Analysis of Circulating Tumor Cells. Methods Mol. Biol..

[20] Malihi P.D., Morikado M., Welter L., Liu S.T., Miller E.T., Cadaneanu R.M., Knudsen B.S., Lewis M.S., Carlsson A., Velasco C.R. (2018). Clonal diversity revealed by morphoproteomic and copy number profiles of single prostate cancer cells at diagnosis. Converg. Sci. Phys. Oncol..

[21] Carlsson A., Kuhn P., Luttgen M.S., Keomanee-Dizon K., Troncoso P., Corn P.G., Kolatkar A., Hicks J.B., Logothetis C.J., Zurita A.J. (2017). Paired High-Content Analysis of Prostate Cancer Cells in Bone Marrow and Blood Characterizes Increased Androgen Receptor Expression in Tumor Cell Clusters. Clin. Cancer Res..

[22] Ruiz C., Li J., Luttgen M.S., Kolatkar A., Kendall J.T., Flores E., Topp Z., Samlowski W.E., McClay E., Bethel K. (2015). Limited genomic heterogeneity of circulating melanoma cells in advanced stage patients. Phys. Biol..

[23] Gerdtsson A., Setayesh S., Malihi P., Ruiz C., Carlsson A., Nevarez R., Matsumoto N., Gerdtsson E., Zurita A., Logothetis C. (2021). Large Extracellular Vesicle Characterization and Association with Circulating Tumor Cells in Metastatic Castrate Resistant Prostate Cancer. Cancers.

[24] Chai S., Matsumoto N., Storgard R., Peng C.-C., Aparicio A., Ormseth B., Rappard K., Cunningham K., Kolatkar A., Nevarez R. (2021). Platelet-Coated Circulating Tumor Cells Are a Predictive Biomarker in Patients with Metastatic Castrate-Resistant Prostate Cancer. Mol. Cancer Res..

[25] Shishido S.N., Sayeed S., Courcoubetis G., Djaladat H., Miranda G., Pienta K.J., Nieva J., Hansel D.E., Desai M., Gill I.S. (2022). Characterization of Cellular and Acellular Analytes from Pre-Cystectomy Liquid Biopsies in Patients Newly Diagnosed with Primary Bladder Cancer. Cancers.

[26] Spearman C. (1987). The Proof and Measurement of Association between Two Things. Am. J. Psychol..

[27] Mann H.B., Whitney D.R. (1947). On a Test of Whether one of Two Random Variables is Stochastically Larger than the Other. Ann. Math. Stat..

[28] Wilcoxon F. (1946). Individual Comparisons of Grouped Data by Ranking Methods. J. Econ. Èntomol..

[29] Van der Maaten L., Hinton G. (2008). Visualizing data using t-SNE. J. Mach. Learn. Res..

[30] Pedregosa F., Varoquaux G., Gramfort A., Michel V., Thirion B., Grisel O., Blondel M., Prettenhofer P., Weiss R., Dubourg V. (2011). Scikit-learn: Machine learning in Python. J. Mach. Learn. Res..

[31] Ward J.H. (1963). Hierarchical grouping to optimize an objective function. J. Am. Stat. Assoc..

[32] Ward M.P., Kane L.E., Norris L.A., Mohamed B.M., Kelly T., Bates M., Clarke A., Brady N., Martin C.M., Brooks R.D. (2021). Platelets, immune cells and the coagulation cascade; friend or foe of the circulating tumour cell?. Mol. Cancer.

[33] Foerster B., Moschini M., Abufaraj M., Soria F., Gust K.M., Rouprêt M., Karakiewicz P.I., Briganti A., Rink M., Kluth L. (2017). Predictive and Prognostic Value of Preoperative Thrombocytosis in Upper Tract Urothelial Carcinoma. Clin. Genitourin. Cancer.

[34] Hu L., Su L., Cheng H., Mo C., Ouyang T., Li J., Wang T., Fan Z., Fan T., Lin B. (2021). Single-Cell RNA Sequencing Reveals the Cellular Origin and Evolution of Breast Cancer in BRCA1 Mutation Carriers. Cancer Res..

[35] Dago A.E., Stepansky A., Carlsson A., Luttgen M., Kendall J., Baslan T., Kolatkar A., Wigler M., Bethel K., Gross M. (2014). Rapid Phenotypic and Genomic Change in Response to Therapeutic Pressure in Prostate Cancer Inferred by High Content Analysis of Single Circulating Tumor Cells. PLoS ONE.

[36] Shishido S.N., Welter L., Rodriguez-Lee M., Kolatkar A., Xu L., Ruiz C., Gerdtsson A.S., Restrepo-Vassalli S., Carlsson A., Larsen J. (2020). Preanalytical Variables for the Genomic Assessment of the Cellular and Acellular Fractions of the Liquid Biopsy in a Cohort of Breast Cancer Patients. J. Mol. Diagn..

[37] Welter L., Xu L., McKinley D., Dago A.E., Prabakar R.K., Restrepo-Vassalli S., Xu K., Rodriguez-Lee M., Kolatkar A., Nevarez R. (2020). Treatment response and tumor evolution: Lessons from an extended series of multianalyte liquid biopsies in a metastatic breast cancer patient. Cold Spring Harb. Mol. Case Stud..

[38] Shishido S.N., Masson R., Xu L., Welter L., Prabakar R.K., Souza A.D., Spicer D., Kang I., Jayachandran P., Hicks J. (2022). Disease characterization in liquid biopsy from HER2-mutated, non-amplified metastatic breast cancer patients treated with neratinib. NPJ Breast Cancer.

[39] Birkenkamp-Demtröder K., Christensen E., Nordentoft I.K., Knudsen M., Taber A., Høyer S., Lamy P., Agerbaek M., Jensen J.B., Dyrskjøt L. (2018). Monitoring Treatment Response and Metastatic Relapse in Advanced Bladder Cancer by Liquid Biopsy Analysis. Eur. Urol..

[40] Christensen E., Birkenkamp-Demtröder K., Nordentoft I., Høyer S., van der Keur K., van Kessel K., Zwarthoff E., Agerbaek M., Ørntoft T.F., Jensen J.B. (2017). Liquid Biopsy Analysis of FGFR3 and PIK3CA Hotspot Mutations for Disease Surveillance in Bladder Cancer. Eur. Urol..

[41] Patel K., Van Der Vos K.E., Smith C.G., Mouliere F., Tsui D., Morris J., Chandrananda D., Marass F., Van Den Broek D., Neal D. (2017). Association of Plasma and Urinary Mutant DNA with Clinical Outcomes in Muscle Invasive Bladder Cancer. Sci. Rep..

