# Supplementary material for: Liquid Biopsy Landscape in Patients with Primary Upper Tract Urothelial Carcinoma
Source: Cancers (Basel). 2022 Jun 18;14(12):3007. doi: 10.3390/cancers14123007 (PMC9221424; doi:10.3390/cancers14123007)
Supplement: Supplementary file 1 [file cancers-14-03007-s001.zip › cancers-1744971-supplementary Table S1.pdf]

Table S1. Enumeration/mL of rare events by channel-type classification ordered by degree of statistical significance between UTUC and ND samples.

	ND		UTUC	
**Characterization**	**Average ± STD**	**Median (range)**	**Average ± STD**	**Median (range)**
Total Rare Events	47.86 ± 29.45	38.5 (4.39-141.55)	234.88 ± 169.65	178.23 (25.66-779.8)
Total Rare Cells	43.21 ± 27.89	34.46 (4.39-137.03)	219.83 ± 169.73	169.85 (18.62-771.63)
Vim|CD45/CD31	7.12 ± 8.28	4.79 (0-34)	89.47 ± 118.03	48.4 (5.39-485.75)
Vim only	9.99 ± 10.5	6.22 (0-47.04)	54.5 ± 56.25	27.54 (1.56-207.76)
Total LEV's	4.65 ± 5.62	3.34 (0-27.91)	15.05 ± 14.12	8.33 (2.97-54.94)
CK|Vim|CD45/CD31	11.03 ± 12.94	5.98 (0-47.48)	51.1 ± 57.03	27.3 (4.47-179.08)
Total CK+	18.96 ± 19.06	12.39 (0-83.24)	60.37 ± 61.53	37.91 (7.06-186.59)
CK only LEVs	3.42 ± 4.41	2.22 (0-19.26)	10.36 ± 14	4.34 (0-54.94)
CD45/CD31	2.08 ± 3.08	1.08 (0-14.32)	4.37 ± 5.86	2.68 (0-23.27)
DAPI only	5.05 ± 6.75	3.76 (0-32.52)	11.12 ± 12.73	7.58 (0-41.7)
CK|CD45/CD31 LEVs	1.01 ± 1.91	0 (0-9.73)	4.05 ± 7.38	1.04 (0-25.08)
Epi.CTCs	0.35 ± 0.85	0 (0-4.87)	1.3 ± 2.04	0 (0-5.9)
CK|Vim|CD45/CD31 LEVs	0.11 ± 0.42	0 (0-2.32)	0.44 ± 0.82	0 (0-2.64)
CK|Vim LEVs	0.1 ± 0.42	0 (0-2.05)	0.2 ± 0.48	0 (0-1.47)
CK|CD45/CD31	6.06 ± 7	4.75 (0-30.89)	5.73 ± 4.89	4.54 (0-18.79)
Mes.CTCs	1.52 ± 3.41	0.58 (0-22.47)	2.24 ± 4.24	0 (0-17)
